# Suprachoroidal triamcinolone versus posterior subtenon triamcinolone either alone or formulated in the management of diabetic macular edema

**DOI:** 10.1007/s10792-023-02856-2

**Published:** 2023-09-12

**Authors:** Ehab Tharwat, Riad Elzaher Hassan Ahmed, Basheer Eltantawy, Ezzeldin Ramadan Ezzeldin, Akram Fekry Elgazzar

**Affiliations:** Department of Ophthalmology, Faculty of Medicine, Al-Azhar University, Damietta, Egypt

**Keywords:** Triamcinolone acetonide (TA), Diabetic retinopathy (DR), Diabetes mellitus (DM), Diabetic macular edema (DME)

## Abstract

**Purpose:**

This study aims to compare posterior subtenon triamcinolone acetonide injection either formulated or alone versus suprachoroidal triamcinolone in the management of diabetic macular edema.

**Methods:**

This study is a prospective interventional study that included 75 patients, divided into three groups, each group with 25 patients. Group I received a combination of triamcinolone acetonide (TA) (40 mg) and VISCOAT, which is a combination of sodium chondroitin sulfate (20 mg) and sodium hyaluronate (15 mg). The injection was done in the posterior subtenon space using the NAGATA cannula. Group II received TA (40 mg) in the posterior subtenon space. Group III underwent an injection of 4 mg/100µl of TA in the supra choroidal space.

**Results:**

We found a statistically significant difference between the three studied groups regarding BCVA (*P* = 0.001) and CMT at six months postoperative (*P* = 0.001) with the highest median BCVA and lowest median CMT observed in the formulated TA group.

**Conclusion:**

We concluded that early treatment of DME by formulated TA is better than TA alone, and suprachoroidal TA in the form of increasing the BCVA and decreasing the CMT without any elevation of IOP.

*Trial registration number* NCT05464953.

*Date of registration* 17/7/2022 (retrospectively registered).

## Introduction

There are multiple diabetic complications, However, DR is considered the most common one [1]. Pathogenesis of DR secondary to diabetes mellitus (DM) is thought to be due that the DM weakening the retina's capillary wall, leading to microaneurysms (MA) formation, which may rupture, and form dot-blot hemorrhages. Also, diabetic hyperglycemia upregulates the vascular endothelial growth factor (VEGF), resulting in capillary occlusions, and small infarctions appearing as fluffy white patches called cotton wool spots [2].

DR is characterized by micro-aneurysms formation, neovascularization, retinal hemorrhages, and exudates resulting in vitreous hemorrhage, tractional retinal detachment, and macular edema [3]. Macular edema is a significant complication for DR. In macular edema, fluid can accumulate in intracellular space, defined as cytotoxic edema, or extracellular space, defined as vasogenic edema. In diabetic macular edema (DME), the cytotoxic form occurs early due to an increased level of sorbitol, lactate, and phosphatase resulting from hyperglycemia. However, the vasogenic condition occurs late due to increased levels of VEGF, nitrous oxide, and other free radicals, which damage the blood-retinal barrier [4].

There are different treatment modalities for macular edema such as; anti-VEGF injection and LASER therapy [5]. Although anti-VEGF is more effective than TA in treating DME, many patients still have residual macular edema even after multiple anti-VEGF injections, which predisposes those patients to a risk of vision loss [6, 7]. Also, anti-VEGF may be contraindicated in some cases such as in pregnant females or cases with epiretinal membranes [8]. So, those patients may need alternatives such as triamcinolone acetonide (TA) injection.

TA is a corticosteroid that has antiangiogenic and anti-inflammatory effects. It can inhibit the proinflammatory cytokines and VEGF expressions, improving visual acuity and decreasing central macular thickness (CMT) in patients with macular edema [9]. The injection of intravitreal triamcinolone acetonide (IVTA) is beneficial. However, it has adverse effects such as cataracts, increased intraocular pressure (IOP), sterile pseudo-endophthalmitis, and endophthalmitis [10]. We can avoid these complications by injecting the triamcinolone in the posterior subtenon or suprachoroidal spaces, which was safer but less effective than IVTA [11, 12].

To increase triamcinolone efficacy, our study hypothesizes that adding sodium hyaluronate and chondroitin sulfate to triamcinolone increases its viscosity, prolonging its contact with the sclera and increasing its diffusion through the scleral barrier [13].

So, this study aims to compare the posterior subtenon triamcinolone acetonide (PSTA) injection either alone or formulated versus suprachoroidal triamcinolone in the management of diabetic macular edema.

## Patient and method

### Study populations

This prospective interventional study was done between January 2020 and April 2022 in the ophthalmology department, at Al-Azhar University, Damietta, Egypt. With a power of 80%, a sample size of 75 patients was calculated. Our research adhered to the principles of the Helsinki Declaration. We included the patients after taking the informed consent. Our data was protected confidentially. We recruited the patient according to the following criteria:

*The Inclusion Criteria include* (1) Diminution of vision due to diabetic macular edema. (2) Central macular thickness ≥ 250 µ.

*The exclusion criteria include* (1) refuse to participate, (2) RVO secondary to ischemia, (3) previous laser therapy, (4) history of ocular diseases such as glaucoma, and cataracts (5) the previous injection of anti-VEGF or steroids or any eye surgery before the study by less than three months, (6) cardiac and respiratory comorbidities, (7) any contraindication to TA such as allergy.

### Data collection

Complete medical history and physical examination were made during enrollment. Comprehensive ophthalmic examinations were done before injection, including; best-corrected visual acuity (BCVA), which was measured by Snellen's chart on a chart projector; Intraocular pressure (IOP), which was measured by applanation tonometer; CMT, which was measured by optical coherence tomography (OCT), and fundus examination using non-contact VOLK +90D lens.

### Drug preparation and route of administration

Patients were divided into three groups; group 1 took the formulated posterior subtenon triamcinolone (TA and VISCOAT), group 2 took the posterior subtenon triamcinolone alone, and Group 3 took the suprachoroidal TA. For all groups, the conjunctiva was anesthetized first by Benoxinate 0.4% drops, then by a soaked microsponge in the lower fornix for five minutes. Standard sterilization by 5% povidone-iodine lid swabbing and instillation of 5% povidone-iodine in the conjunctival sac was done.

Superior-temporal subconjunctival anesthesia (2% lidocaine) was done then; a small tenon and conjunctival incisions were made to the bare sclera (7 mm posterior and superior temporal to the limbus) in groups 1 and 2; then the patient in group 1 received a combination of TA (40 mg) and VISCOAT. This VISCOAT consisted of a combination of sodium chondroitin sulfate (20 mg) and sodium hyaluronate (15 mg). This suspension was mixed in a 5 ml syringe by shaking well for 2 min till mixing well, and no fluid level was seen. The injection was done in the posterior subtenon space using a NAGATA cannula.

The patients in group 2 underwent a posterior subtenon injection of 40 mg TA alone. To avoid injection reflux from the opening, we injected it using a cannula by applying pressure on the start of the conjunctiva with a cotton tip; then, we removed the cannula very slowly and closed the conjunctiva with diathermy. The patients in group 3 underwent an injection of 4 mg/100µl of TA in the suprachoroidal space using a microneedle (Clearside Biomedical, Inc) insertion at the pars plana.

All injections were done in the operating rooms under complete aseptic conditions. Antibiotic eye drops and nonsteroidal anti-inflammatory eye drops were prescribed one week after injection. All cases received a macular grid and focal argon laser one month after injection.

### Follow-up

Patients were examined at months one, three, and six after injection. The complete ophthalmic assessment was done during follow-up as those at baseline. Reinjection was indicated if the CMT was more than 300 µm.

### Statistical analysis

We analyzed our data by using SPSS version 25 (IBM Corp., Armonk., NY., USA). The continuous data were first examined for normality using the Shapiro–Wilk test. We presented them as mean and standard deviations if they were parametric. If the continuous data were not parametric, we presented them as the median and interquartile range (IQR). Kruskal–Wallis test was used to compare three groups and followed by Mann–Whitney *U*-test to compare every two groups. Additionally, within-group comparisons were made using the Friedman and Wilcoxon signed-rank tests.

## Results

Seventy-five patients with DME (*n* = 75 eyes) were enrolled in this study, they were divided into three groups, each group with 25 patients. The demographic characteristics of the patients are shown in Table 1. Regarding BCVA, by comparing the three studied groups throughout the follow-up periods, we found a statistically significant difference among them at one month and six months post-operative (*P* < 0.001). This difference disappeared at three months post-operative (*P* < 0.3) with the highest median BCVA observed in formulated TA group (Table 2).

**Table 1:** Demographic characteristics of the studied patients

Variables	Total (*n* = 75 eyes)	TA alone (*n* = 25 eyes)	Formulated TA (*n* = 25 eyes)	Suprachoroidal (*n* = 25 eyes)	*P* value^a^
Age (years) (Mean ± SD)	55.2 ± 5	56.1 ± 5.2	54.7 ± 5	54.9 ± 4.8	0.57^a^
*Gender* *N* (%)					
Male	28 (37.3%)	9 (36%)	10 (40%)	9 (36%)	0.94^b^
Female	47 (62.7%)	16 (64%)	15 (60%)	16 (64%)	

Data represented as median (IQR)

^a^One way ANOVA

^b^Chisquare test

**Table 2:** Comparison of BCVA among three studied groups over the follow-up periods

BCVA (LogMar)	TA alone (*n* = 25 eyes)	Formulated TA (*n* = 25 eyes)	Suprachoroidal (*n* = 25 eyes)	*P* value^a^	*P* value^b^ between groups
Pre-intervention	0.5(0.5–0.60)	0.4(0.4–0.50)	0.50(0.40–0.6)	< 0.002*	P1 = 0.001* P2 < 0.4 P3 < 0.001*
Post-1 month	0.6 (0.5–0.60)	0.5 (0.4–0.50)	0.60(0.60–0.80)	< 0.001*	P1 = 0.001* P2 < 0.001* P3 < 0.001*
Post-3 months	0.6 (0.55–0.70)	0.6(0.5–0.70)	0.70(0.50–0.70)	< 0.3*	P1 = 0.417 P2 < 0.1 P3 < 0.6
Post-6 months	0.7 (0.60–0.70)	0.8 (0.7–0.80)	0.60(0.50–0.70)	< 0.001*	P1 = 0.001* P2 < 0.3 P3 = 0.001*

Data represented as median (IQR)

^a^Kruskal–Wallis test

^b^Mann–Whitney *U*-test

P1: Comparison between TA alone group and formulated TA group

P2: Comparison between TA alone group and suprachoroidal group

P3: Comparison between formulated TA group and suprachoroidal group

*Statistically significant at *P* < 0.05

In both groups who received the PSTA, there was no significant elevation in the BCVA at one month post-intervention (*P* > 0.05); however, there was a significant elevation at three and six months post-operative (*P* = 0.01). Also, we found a considerable difference regarding BCVA between 1 month post-intervention and three months and six months post-intervention (*P* = 0.01). In the suprachoroidal group, we found a significant elevation in BCVA at 1 month and three months, and six months post-intervention (*P* = 0.001). However, we found no significant difference between 1-month post-intervention and three months and six months post-intervention (*P* = 0.3; *P* = 0.07) (Table 3).

**Table 3:** Comparison of BCVA in each of the three studied groups throughout the follow-up period

BCVA (LogMar)	Pre-intervention	Post-1 month	Post-3 months	Post-6 months	Overall *P* value^a^
TA alone	0.5 (0.5–0.60)	0.6(0.5–0.6)	0.6(0.55–0.7)	0.7 (0.6–0.70)	0.0001*
	P1^b^	0.61	0.01	0.001	
		P2^b^	0.01	0.001	
			P3^b^	0.01	
Formulated TA	0.4(0.4–0.5)	0.5 (0.4–0.5)	0.6(0.5–0.7)	0.8 (0.7–0.8)	< 0.001*
	P1^b^	0.1	< 0.001**	< 0.001**	
		P2^b^	< 0.001**	< 0.001**	
			P3^b^	< 0.001**	
Suprachoroidal	0.5(0.7–1.00)	0.6(0.6–0.9)	0.7(0.5–0.9)	0.6(0.5–0.9)	< 0.0001*
	P1^b^	< 0.001**	< 0.002**	< 0.01**	
		P2^b^	< 0.3	< 0.07	
			P3^b^	< 0.1	

Data represented as median (range)

^a^Friedman test

^b^Wilcoxon signed ranks test

P1 = Pairwise comparison between baseline and post-1 month, post-3 months, and post-6 months

P2 = Pairwise comparison between a post-1 month and post-3 months, and post-6 months

P3 = Pairwise comparison between post-3 months and post-6 months

*Statistically significant at *P* < 0.05

**Statistically significant at *P* < 0.05

**Fig. 1:**
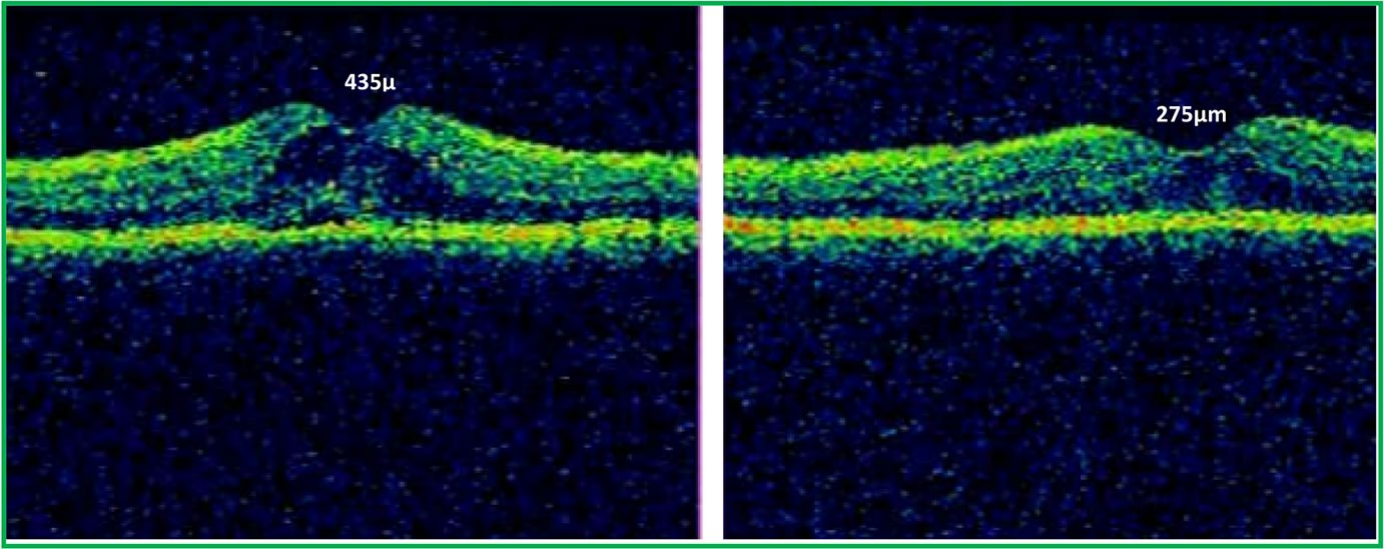
Pre and Post Formulated PSTA injection SD-OCTs showing marked reduction in CMT

Table 4 shows statistically significant differences between the three studied groups post one month, three months, and six months of intervention regarding CMT (*P* = 0.001). After six months of intervention, CMT was significantly lower in the formulated TA group than in the suprachoroidal and TA-alone groups (*P* < 0.001) (figure 1). Also, we found a significant decrease in the CMT at all follow-up periods in all groups (*P* < 0.001). In both groups who received PSTA, we found a significant difference between CMT at one month, three months, and six months with the lowest reduction observed at six months in the formulated group (*P* < 0.001). However, in the suprachoroidal group, there was no difference between one month and three months, and six months post-operative (*P* = 0.3; *P* = 0.4) (Table 5). In terms of the IOP, it was within the normal range in all study groups during all follow-up periods (Tables 6, 7). Regarding the reinjection found that the least number of patients who required re-injection was in the formulate PSTA group compared to the other two groups.

**Table 4:** Comparison of CMT among three studied groups over the follow-up periods

CMT(μm)	TA alone (*n* = 25 eyes)	Formulated TA (*n* = 25 eyes)	Suprachoroidal (*n* = 25 eyes)	*P* value^a^	*P* value^b^ between groups
Pre-intervention	375.60(313–451)	591 (523–629)	427 (386–481.5)	< 0.001*	P1 < 0.001* P2 = 0.016* P3 < 0.001*
Post-1 month	354(292–389.5)	361 (329–406.5)	284(264.5–313)	< 0.001*	P1 = 0.22 P2 = 0.001* P3 = 0.001*
Post-3 months	301(275–336.5)	267 (253.5–278)	292 (270.5–360)	< 0.001*	P1 = 0.001* P2 = 0.8 P3 = 0.001*
Post-6 months	294(269–313.5)	228 (209.5–241)	276 (252.5–303)	< 0.001*	P1 < 0.001* P2 = 0.15 P3 = 0.001*

Data represented as median (range)

^a^Kruskal–Wallis Test

^b^Mann–Whitney *U*-test

P1: Comparison between TA alone group and formulated TA group

P2: Comparison between TA alone group and suprachoroidal group

P3: Comparison between formulated TA group and suprachoroidal group

*Statistically significant at *P* < 0.05

**Table 5:** Comparison of CMT in each of the three studied groups throughout the follow-up period

CMT (μm)	Pre-intervention	Post-1 month	Post-3 months	Post-6 months	Overall *P* value^a^
TA alone	375.60(313–451)	354(292–389.5)	301(275–336.5)	294(269–313.5)	< 0.001*
	P1^b^	0.02	0.001*	0.001*	
		P2^b^	< 0.001*	< 0.001*	
			P3^b^	0.1	
Formulated TA	591 (523–629)	361 (329–406.5)	267 (253.5–278)	228 (209.5–241)	< 0.001*
	P1^b^	< 0.001*	< 0.001*	< 0.001*	
		P2^b^	< 0.001*	< 0.001*	
			P3^b^	< 0.001*	
Suprachoroidal	427 (386–481.5)	284(264.5–313)	292 (270.5–360)	276 (252.5–303)	< 0.001*
	P1^b^	< 0.001*	< 0.001*	< 0.001*	
		P2^b^	< 0.3	< 0.0.4	
			P3^b^	< 0.001*	

Data represented as median (range)

^a^Friedman test

^b^Wilcoxon signed ranks test

P1 = Pairwise comparison between baseline and post-1 month, post-3 months, and post-6 months

P2 = Pairwise comparison between a post-1 month and post-3 months, and post-6 months

P3 = Pairwise comparison between post-3 months and post-6 months

*Statistically significant at *P* < 0.05

**Table 6:** Comparison of IOP among three studied groups over the follow-up periods

IOP (mmHg)	TA alone (*n* = 25 eyes)	Formulated TA (*n* = 25 eyes)	Suprachoroidal (*n* = 25 eyes)	*P* value^a^	*P* value^b^ between groups
Pre-intervention	13 (12–14)	15 (13.5–17)	13 (12–15)	< 0.001*	P1 = 0.001* P2 < 0.4 P3 < 0.007*
Post-1 month	13 (12–13.5)	15 (13–16)	13 (12–14)	< 0.001*	P1 = 0.001* P2 < 0.23 P3 < 0.004*
Post-3 months	13 (12–14)	15 (13–16)	13 (12–14)	< 0.005*	P1 = 0.002 P2 < 0.13 P3 < 0.02*
Post-6 months	13 (12–14)	15 (13.5–16)	13 (12–15)	< 0.003*	P1 = 0.001* P2 < 0.3 P3 = 0.02*

Data represented as median (IQR)

^a^Kruskal–Wallis Test

^b^Independent t test

P1: Comparison between TA alone group and formulated TA group

P2: Comparison between TA alone group and suprachoroidal group

P3: Comparison between formulated TA group and suprachoroidal group

*Statistically significant at *P* < 0.05

**Table 7:** Comparison of IOP in each of the three studied groups throughout the follow-up period

IOP (mmHg)	Pre-intervention	Post-1 month	Post-3 months	Post-6 months	Overall *P* value^a^
TA alone	13 (12–14)	13 (12–13.5)	13 (12–14)	13 (12–14)	0.1
	P1^b^	0.04*	0.2	1	
		P2^b^	0.4	0.03*	
			P3^b^	0.23	
Formulated TA	15 (13.5–17)	15 (13–16)	15 (13–16)	15 (13.5–16)	< 0.009*
	P1^b^	0.008*	< 0.004**	< 0.05**	
		P2^b^	1	< 0.2	
			P3^b^	< 0.3	
Suprachoroidal	13 (12–15)	13 (12–14)	13 (12–14)	13 (12–15)	< 0.01*
	P1^b^	< 0.007*	< 0.2	1	
		P2^b^	< 0.1	< 0.003*	
			P3^b^	< 0.31	

Data represented as median (range)

^a^Friedman test

^b^Wilcoxon signed ranks test

P1 = Pairwise comparison between baseline and post-1 month, post-3 months, and post-6 months

P2 = Pairwise comparison between a post-1 month and post-3 months, and post-6 months

P3 = Pairwise comparison between post-3 months and post-6 months

*Statistically significant at *P* < 0.05

**Statistically significant at *P* < 0.05

## Discussion

### Summary of the main findings

Our study showed that injection of formulated PSTA or PSTA alone or suprachoroidal TA effectively treats diabetic macular edema by reducing the CMT and increasing the BCVA over a 6-month follow-up period without any rise in IOP. Compared to the baseline, there was a significant rise in BCVA and a significant decrease in CMT at month 1, month 3, and month 6 in each group. However, by comparing the three studied groups, we found a statistically significant difference between them regarding BCVA and CMT with the highest BCVA and lowest CMT being observed in the formulated PSTA group six months postoperative. We also found that the least number of patients who required re-injection was in the formulate PSTA group compared to the other two groups.

### Explanation of the main finding of the study

The rationale for using the TA in treating diabetic macular edema is that many patients still have residual macular edema even after multiple anti-VEGF injections. Also, these various injections need the high economic state of the patients. Those factors may predispose the patients to vision loss. So, TA may solve these problems and protect those patients from vision [7] TA is a corticosteroid that has an anti-inflammatory effect, so it can inhibit some factors that enter the pathogenesis of DME, such as VEGF, improving BCVA and decreasing CMT [9, 14, 15]. Formulated PSTA is better than none formulated PSTA and suprachoroidal TA as the addition of sodium hyaluronate and chondroitin sulfate to TA increases the triamcinolone viscosity, prolongs its contact with the sclera and increases its diffusion through the scleral barrier making its efficacy better [13].

### Agreement and disagreement with previous studies

We did not find a previous study comparing the PSTA either formulated or alone versus suprachoroidal TA. However, a survey by Veritti et al. [13] treated 18 patients with diabetic macular edema by posterior juxta scleral injection of the formulated TA, resulting in an improvement in the BCVA in 90% of the patients. Although the different injection sites between them and us, this result agrees with our findings and strengthens our hypothesis that adding sodium hyaluronate and chondroitin sulfate increases the efficacy of TA in treating DME. Another study was done by Acharya [16], who found that PSTA injection is effective in treating ME secondary to different eye conditions, which is consistent with our study findings. However, he reported an elevation in the IOP of more than 21 mmHg in four eyes after the injection by one week, which is inconsistent with our results. This elevation in the IOP may be due to a higher IOP before injection or a large steroid dose. We also found that suprachoroidal TA is effective for treating DME, which agrees with the finding of Tayyab et al. [12], who included 24 patients with DME and found a significant rise in the BCVA and decrease in the Central Subfield Thickness over three months follow-up.

### Significance of our study

Our study introduces evidence of the efficacy of PSTA either alone or formulated and suprachoroidal TA in treating DME. We also submit proof that formulated TA is better than the none formulated TA and suprachoroidal TA in treating the DME.

### Strength points and limitations

It is the first study on Egyptian populations and also worldwide to compare the efficacy of Posterior Subtenon Triamcinolone either formulated or alone versus suprachoroidal TA in treating DME. The main limitation of our study is the small sample size and short follow-up period.

### Recommendations for future research and clinical practice

Multicentre studies with larger samples and longer follow-up periods are required to show us that this improvement in vision and CMT is transient or prolonged-lasting.

### Authors’ conclusions

We concluded that early treatment of DME by formulated TA is more effective than TA alone, and suprachoroidal TA in the form of increasing the BCVA and decreasing the CMT without any elevation of IOP.
